# Supplementary material for: Expression and Prognostic Value of a Novel B7-H3 (CD276) Antibody in Acute Myeloid Leukemia
Source: Cancers (Basel). 2024 Jul 4;16(13):2455. doi: 10.3390/cancers16132455 (PMC11240323; doi:10.3390/cancers16132455)
Supplement: Supplementary file 1 [file cancers-16-02455-s001.zip › cancers-3069204-supplementary/Figure S1.pdf]

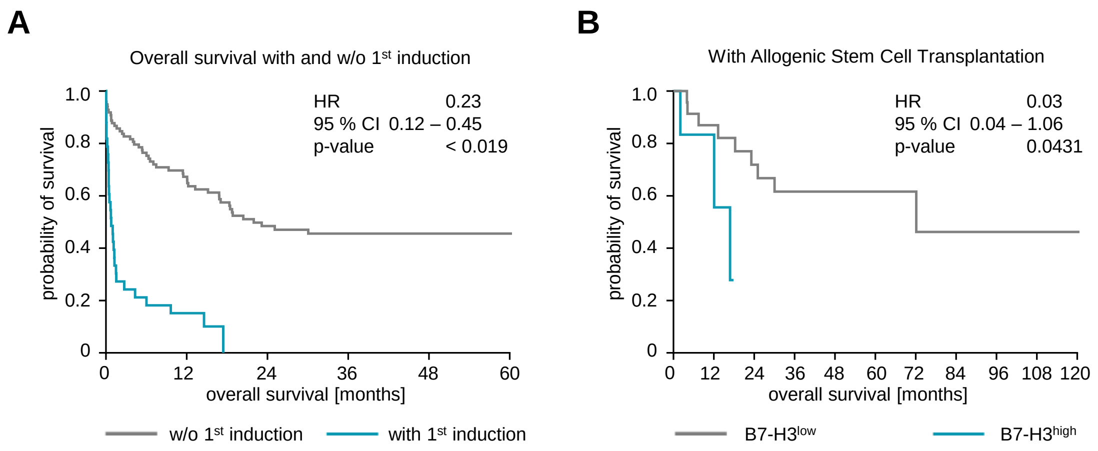

Supplementary Figure S1: Overall Survival

A Overall survival according to with and without 1st induction.

B Overall survival of patients receiving allogenic stem cell transplantation.
