# Supplementary material for: Expression and Prognostic Value of a Novel B7-H3 (CD276) Antibody in Acute Myeloid Leukemia
Source: Cancers (Basel). 2024 Jul 4;16(13):2455. doi: 10.3390/cancers16132455 (PMC11240323; doi:10.3390/cancers16132455)
Supplement: Supplementary file 1 [file cancers-16-02455-s001.zip › cancers-3069204-supplementary/Tables S1 and S2.docx]

### Supplementary Table S1: Patients treatment regimes, - responses and genetic characteristics

| **Induction** | **n [77]** | **% of known** |
| --- | --- | --- |
| no | 22 | 29.0 |
| yes | 55 | 71.0 |
| **1st induction therapy regimen:** | n [55] |  |
| Anthracycline based n [46]: |  |  |
| DA | 21 | 38.18 |
| DA + Midostaurin | 5 | 9.09 |
| ICE | 2 | 3.63 |
| ICE + Midostaurin | 4 | 7.27 |
| Other | 14 | 25.45 |
| Non-anthracycline based | 6 | 10.91 |
| Unknown | 3 | 5.45 |
| **Consolidation** | n [50] |  |
| allo-HCT | 29 | 58.0 |
| HDAC | 12 | 24.0 |
| IDAC | 4 | 8.0 |
| LDAC | 2 | 4.0 |
| other | 2 | 4.0 |
| unknown | 1 | 2.0 |
| **allo-HCT** |  |  |
| no | 47 | 62.0 |
| yes | 29 | 38.0 |
| unknown | 1 | - |
| **Response to 1^st^ Induction** |  |  |
| CR(i) | 31 | 71.1 |
| PR | 13 | 28.9 |
| unknown | 10 | - |
| **Karyotype** |  |  |
| normal | 40 | 57.1 |
| < 3 aberrations | 23 | 32.9 |
| complex aberrations | 7 | 10.0 |
| unknown | 7 | - |
| **PML/RARA t(15;17)** |  |  |
| wildtype | 65 | 91.6 |
| aberrant | 6 | 8.5 |
| unknown | 6 | - |
| **RUNX1/RUNX1T1 t(8;21)** |  |  |
| wildtype | 67 | 98.5 |
| aberrant | 1 | 1.5 |
| unknown | 9 | - |
| **CBFB/MYH11 inv(16)** |  |  |
| wildtype | 67 | 95.7 |
| aberrant | 3 | 4.3 |
| unknown | 7 | - |
| **FLT3-ITD** |  |  |
| wildtype | 40 | 58.8 |
| mutated | 28 | 41.2 |
| S | 9 | - |
| **NPM1** |  |  |
| wildtype | 39 | 58.2 |
| mutated | 28 | 41.8 |
| unknown | 10 | - |
| **CEBPA** |  |  |
| wildtype | 54 | 91.5 |
| mutated | 5 | 8.5 |
| unknown | 18 | - |
| **DA:** daunorubicin, cytarabine, **ICE:** idarubicin, cytarabine, etoposide, **HDAC:** high dose cytarabine; **IDAC:** intermediate dose cytarabine; **LDAC:** low dose cytarabine; **t()**: translocation, **inv()**: inversion, **allo-HCT:** allogeneic hematopoietic-cell transplantation, **CR(i)**: complete response (with incomplete hematologic recovery), **PR**: partial response, Response classification according to ELN 2022 (7). | | |

### Supplementary Table S2: Patients genetic characteristics according to B7-H3^low^ and B7-H3^high^

|  | **B7-H3 low (SFI < 4.45)**  (n = 58) | | **B7-H3 high (SFI** ≥ **4.45)**  (n = 19) | | **p-value** |
| --- | --- | --- | --- | --- | --- |
| **Karyotype** | **n** | **% within group** | **n** | **% within group** |  |
| normal | 32 | 60.4 | 8 | 47.1 |  |
| < 3 aberrations | 18 | 34.0 | 5 | 29.4 |  |
| complex aberrations | 3 | 5.7 | 4 | 23.5 | 0.203^‡^ |
| unknown | 5 | - | 2 | - |  |
| **PML/RARA t(15;17)** |  |  |  |  |  |
| wildtype | 50 | 92.6 | 15 | 88.2 |  |
| aberrant | 4 | 7.4 | 2 | 11.8 | 0.751^‡^ |
| unknown | 4 | - | 2 | - |  |
| **RUNX1/RUNX1T1 t(8;21)** |  |  |  |  |  |
| wildtype | 51 | 100.0 | 16 | 94.1 |  |
| aberrant | 0 | 0.0 | 1 | 5.9 | 0.212^‡^ |
| unknown | 7 | - | 2 | - |  |
| **CBFB/MYH11 inv(16)** |  |  |  |  |  |
| wildtype | 50 | 94.3 | 17 | 100.0 |  |
| aberrant | 3 | 5.7 | 0 | 0.0 | 0.589^‡^ |
| unknown | 5 | - | 2 | - |  |
| **FLT3-ITD** |  |  |  |  |  |
| wildtype | 30 | 58.8 | 10 | 58.8 |  |
| mutated | 21 | 41.2 | 7 | 41.2 | 0.984^‡^ |
| unknown | 7 | - | 2 | - |  |
| **NPM1** |  |  |  |  |  |
| wildtype | 29 | 56.9 | 10 | 62.5 |  |
| mutated | 22 | 43.1 | 6 | 37.5 | 0.848^‡^ |
| unknown | 7 | - | 3 | - |  |
| **CEBPA** |  |  |  |  |  |
| wildtype | 40 | 88.9 | 14 | 100.0 |  |
| mutated | 5 | 11.1 | 0 | 0.0 | 0.411^‡^ |
| unknown | 13 | - | 5 | - |  |
| ^‡^Pearson's chi-squared test  **t()**: translocation, **inv()**: inversion | | | | | |
